# Pseudoacromegaly with acromegalic features in radiography

**DOI:** 10.1002/ccr3.4095

**Published:** 2021-05-15

**Authors:** Koichiro Yamamoto, Kosuke Oka, Hiroyuki Honda, Kou Hasegawa, Fumio Otsuka

**Affiliations:** ^1^ Department of General Medicine Okayama University Graduate School of Medicine, Dentistry and Pharmaceutical Sciences Okayama Japan

**Keywords:** acromegaloidism, gigantism, growth hormone, insulin‐like growth factor‐I, pseudoacromegaly

## Abstract

Pseudoacromegaly is a condition characterized by acromegalic physical features without growth hormone excess, for which radiographic observation has seldom been reported. This is a rare case of pseudoacromegaly.

## Graphical abstract

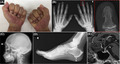

A 64‐year‐old male with medical history of hypertension and past medical history of ureterolithiasis presented with unsteady gait. His height, body weight, and body mass index were 164.5 cm, 74.6 kg, and 27.6 kg/m^2^, respectively. Physical assessment revealed enlargement of his nose, lips, and eyebrow arches and positive fist sign (A). An X‐ray showed cauliflower‐like tufting of the distal phalanxes of his hands (B), enlargement of the frontal sinus (C: *arrowheads*), and heel pad thickness of 22 mm (D: *two‐way arrow*). Serum growth hormone (GH) and insulin‐like growth factor‐I (IGF‐I) were 0.64 ng/mL and 259.00 ng/mL (+2.7 SD), respectively, whereas an oral glucose tolerance test (OGTT) showed significant suppression of GH production (nadir: 0.17 ng/mL). Contrast‐enhanced pituitary magnetic resonance imaging (MRI) showed no tumor (E) (Figure 1).

**FIGURE 1:**
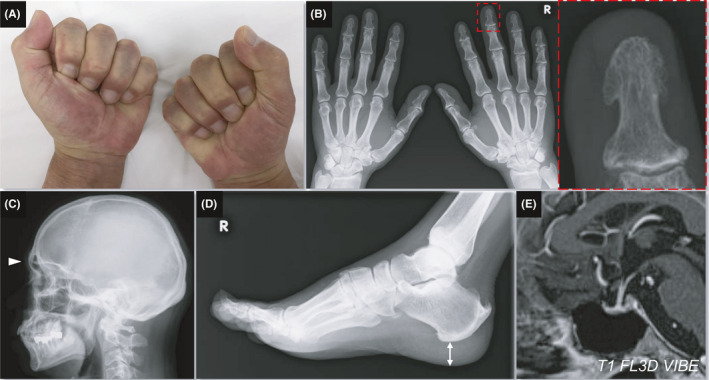
A, Positive fist sign. B, Cauliflower‐like tufting of the distal phalanxes in a hand X‐ray. The dotted line in the left panel corresponds to the outline of the right panel. C, Head X‐ray showed enlargement of the frontal sinus (*arrowhead*). D, Foot X‐ray showed heel pad thickness of 22 mm (*two‐way arrow*). E, Contrast‐enhanced pituitary magnetic resonance imaging showed no tumor

The present case had clinical features resembling acromegaly, but an OGTT and MRI showed that there was no GH‐secreting tumor, suggesting pseudoacromegaly. Pseudoacromegaly or acromegaloidism is a rare syndrome characterized by an acromegalic physical appearance or gigantism without GH/IGF‐I axis abnormalities.^1^, ^2^ An X‐ray revealed acromegalic features in this case,however, results of radiological assessments for acromegaloidism have seldom been reported. Further genetic investigation was deferred due to the lack of the patient's consent. Pseudoacromegaly should be recognized as an important differential diagnosis for patients with acromegaloid characteristics in physical or radiological findings.

## CONFLICT OF INTEREST

The authors declare no conflicts of interest.

## AUTHOR CONTRIBUTIONS

KY: involved in writing the first draft and managed all of the submission process. KO, HH, and KH: contributed to the clinical management of the patient. FO: organized writing the manuscript.

## ETHICAL APPROVAL

Informed consent was obtained from the patient to publish this case report.
